# Managing complexity in the operating room: a group interview study

**DOI:** 10.1186/s12913-020-05192-8

**Published:** 2020-05-19

**Authors:** Camilla Göras, Ulrica Nilsson, Mirjam Ekstedt, Maria Unbeck, Anna Ehrenberg

**Affiliations:** 1 School of Education, Health and Social Studies, Dalarna University, Falun, Sweden; 2 Department of Anesthesia and Intensive Care Unit, Falu Hospital, Falun, Sweden; 3 Center for Clinical Research, Falun, Dalarna Sweden; 4 Department of Neurobiology, Care Sciences and Society, Karolinska Institutet, Stockholm, Sweden; 5 Perioperative Medicine and Intensive Care, Karolinska University Hospital, Stockholm, Sweden; 6 Department of Health and Caring Sciences, Linnaeus University, Kalmar/Växjö, Sweden; 7 Department of Learning, Informatics, Management and Ethics, Karolinska Institutet, Stockholm, Sweden; 8 Acute and Reparative Medicine Theme, Karolinska University Hospital, Stockholm, Sweden

**Keywords:** Complexity, Group interviews, Operating room, Patient safety and work processes

## Abstract

**Background:**

Clinical work in the operating room (OR) is considered challenging as it is complex, dynamic, and often time- and resource-constrained. Important characteristics for successful management of complexity include adaptations and adaptive coordination when managing expected and unexpected events. However, there is a lack of explorative research addressing what makes things go well and how OR staff describe they do when responding to challenges and compensating for constraints. The aim of this study was therefore to explore how complexity is managed as expressed by operating room nurses, registered nurse anesthetists, and surgeons, and how these professionals adapt to create safe care in the OR.

**Method:**

Data for this qualitative explorative study were collected via group interviews with three professional groups of the OR-team, including operating room nurses, registered nurse anesthetists and operating and assisting surgeons in four group interview sessions, one for each profession except for ORNs for which two separate interviews were performed. The audio-taped transcripts were transcribed verbatim and analyzed by inductive qualitative content analysis.

**Results:**

The findings revealed three generic categories covering ways of creating safe care in the OR: *preconditions and resources*, *planning and preparing for the expected and unexpected*, and *adapting to the unexpected*. In each generic category, one sub-category emerged that was common to all three professions: *coordinating and reaffirming information*, *creating a plan for the patient and undergoing mental preparation*, and *prioritizing and solving upcoming problems*, respectively.

**Conclusion:**

Creating safe care in the OR should be understood as a process of planning and preparing in order to manage challenging and complex work processes. OR staff need preconditions and resources such as having experience and coordinating and reaffirming information, to make sense of different situations. This requires a mental model, which is created through planning and preparing in different ways. Some situations are repetitive and easier to plan for but planning for the unexpected requires anticipation from experience. The main results strengthen that abilities described in the theory of resilience are used by OR staff as a strategy to manage complexity in the OR.

## Background

Clinical work in the operating room (OR) is dynamic, and complex, and often time- and resource-constrained [1]. Performing surgical procedures requires, specific technical and cognitive skills from OR staff, such as anticipating patients’ needs, managing changes and handling unexpected events [1, 2]. Increased co-morbidities of patients [3], and pressure for efficiency and productivity [4, 5] are other challenges that may influence the work in the OR. Teams in the OR interact, communicate, adapt, learn and self-organize over time [6, 7] which are common determinants of a complex adaptive system (CAS) [7]. From the perspective of complexity there are different strategies for improving patient safety, from attempting to control complexity to embracing it by encouraging flexible behaviors [8]. Complexity requires to wisely balance thoroughness and control with flexibility and adaptations [9]. The surgical safety checklist [10] is an example of a procedure that structures safe care processes in the OR that lay ground for patient safety which also can include flexibility in the face of unpredictable events. Complexity means that work processes may be disturbed or interrupted by unpredictable events that the OR staff has to adapt to and handle [11]. Adaptations contribute to keeping the system’s performance at an acceptably high level under both ordinary and extraordinary conditions, but can also create high-risk situations [12]. According to the coordination and mobilization of many interdependent processes, support and resources in a CAS are seldom optimal which may produce strain among staff and lead them to develop compensatory strategies [13].

In the attempts to understand and influence how complex systems such as OR works, traditional ways of thinking in forms of linear causality models are insufficient. A ‘system thinking’ approach that consider the flow of interactive activities (e.g. between people, equipment, procedures) and the continuous adjustments needed to cope with system variability can help to improve safety and performance in the daily practice in an OR [12]. Patient safety in the OR should be understood by studying “work-as-done”, which reflects the reality that professionals have to deal with in their everyday clinical work, rather than through the ideal picture of “work-as-imagined” which is often presented in policy documents or action plans [12]. However, a deeper knowledge is needed to understand how “the work is described as being done” in relation to safe care in the OR.

Strategies to cope with and adapt to complexity have been described from the perspective of Resilience engineering, RE [14, 15]. Resilience is defined as the ability of the healthcare system to adjust its functioning prior to, during, or following changes or disturbances, so that required operations can be sustained under expected and unexpected conditions [16]. From a RE perspective, rather than controlling what professionals do, patient safety is strengthened by a systemic capacity which enables professionals to be reflexive, to adapt to changing conditions, and to understand the whole system [14]. Resilient organizations is often described through four abilities: the ability to *respond* to events, to *monitor* ongoing developments, to *anticipate* future threats and opportunities and to *learn* from past failures and successes [16]. Resilience research has shown that ways of managing complexity are also characterized by abilities such as anticipation, sensemaking, trade-offs, and adaptation [17]. Operationalization of resilience in inpatient healthcare is characterized by professionals anticipating and bridging gaps by proactively monitoring and acting on problems [18]. Adaptive coordination, the ability of a team to change its coordination activities in response to unexpected events and varying task characteristics [19], are other cornerstones of effective team performance in complex settings [20]. Preoperative huddles have shown to contribute to improvements in patient safety, communication, and teamwork. Postoperative debriefings after non-routine and routine cases are other strategies that stimulated learning, and improved work processes and teamwork [21]. Behaviors to manage non-routine events in the OR are also described to include task- and information management, teaching, and leadership [19].

To cope with complexity, that is managing expected and unexpected events, resilience has been described to be important. When managing unexpected events in the OR adaptive coordination was described an important skill. However, there is a lack of explorative research addressing what makes things go well and how the OR staff describe they do when responding to challenges and compensating for constraints. This can be understood by describing how health professionals describe that work is done in a clinical setting. Knowing how surgical teams manage complexity will be an important contribution to a deeper understanding of how patient safety is created in a collaborative way in the OR. The aim of this study was therefore to explore how complexity is managed as expressed by operating room nurses (ORNs), registered nurse anesthetists (RNAs), and surgeons, and how these professionals adapt to create safe care in the OR.

## Methods

### Setting and sample

This study employed a qualitative explorative design by using group interviews with OR staff. The interviews were conducted at two central OR departments at one county hospital and one local county hospital in mid-Sweden. Each hospital had one department for day surgery and one central OR department. The central OR department at the local county hospital served both acute and elective surgical and orthopedic patients, whereas the OR department at the county hospital in addition also served gynecological patients. Teams in Swedish ORs commonly comprise six different professionals: ORN, operating surgeon (surgeon), assisting surgeon, circulating nurse (commonly a licensed practical nurse) anesthesiologist and RNA. In Sweden, RNAs are allowed to maintain anaesthesia with direct or indirect supervision of the anesthesiologist [22]. The sample consisted of three professional groups of the OR-team, including ORNs, RNAs and operating and assisting surgeons in four group interview sessions, one for each profession except for ORNs for which two separate interviews were performed. Two ORNs at the county hospital, who participated in the pilot interview, were included, to achieve large enough group sizes. The four groups comprised a convenience sample of professionals who were available to be released from clinical work and who had been employed at the OR for least 6 months. The interviews were conducted at separate occasions divided in groups by professional specialization. The informants’ characteristics are given in Table 1.

**Table 1:** Characteristics of the informants: operating room nurses (ORNs), registered nurse anesthetists (RNAs), and surgeons

Characteristics	ORNs (*n* = 4)	RNAs (*n* = 5)	Surgeons (*n* = 8)	Total (*n* = 17)
Gender, *n*				
Female	4	2	3	9
Male	–	3	5	8
Age, years, mean (range)	52 (37–61)	59 (49–66)	51 (34–67)	54
Years of experience as a specialist, mean (range)	27 (9–38)	22 (15–36)	16 (0–34)	22

### Data collection

Open questions were asked based on an interview guide which had been developed by the researchers. The interview guide was pilot tested and resulted in a minor rearranging of the themes, but no revisions or changes in content were needed. The interview guide consisted of five questions including “Can you tell me how you plan your day at work?”, “Could you tell me about situations when the work proceeded according to plan?”, “Could you tell me about situations when work did not proceed according to plan?”, “What enables and what hinders you from being able to do the work as planned?”, and "Do you ever have to abandon routines. To get permission to conduct the study, information was provided both verbally and in writing to the medical director of the surgical department and nurse managers at the OR department who invited their staff to participate. Those who volunteered gave their written informed consent after receiving verbal and written information including the voluntary nature of participation and the ability to withdraw at any time without further explanation, and confidential treatment of data.

The data were collected during February and April 2018, via scheduled 1-h interviews in an undisturbed and quiet location at the workplace. At the beginning of each session, the moderator and the assistant (i.e., the first and last authors) gave a brief presentation of the study, including the aim of the study and why the participants were selected. The discussions were led by the same moderator (first author) throughout all four interviews. The interviews were audiotaped, and field notes were taken by the assistant. The interviews lasted between 50 and 59 min and were transcribed verbatim.

### Data analysis

The interviews were analyzed by using inductive qualitative content analysis focused on the manifest content [23]. All the authors are registered nurses or RNAs with experience of healthcare and the OR, and all participated throughout the analytical process to identify codes, sub-categories, and generic categories. Transcripts were read thoroughly several times to obtain a sense of the whole. Content that related to the aim of the study was noted first in the margins of the text and then on a coding sheet. The codes were based on similarities and differences and were sorted into sub-categories which were then interpreted and aggregated into broader generic categories. The different steps were discussed within the research team. To maintain consistency, there was a movement back and forth between the transcripts, codes, sub-categories, and generic categories. To reach consensus, the research group independently categorized the codes and discussed the findings several times. The analysis generated three generic categories. An example of the analytical procedure is presented in Table 2.

**Table 2:** Examples of transcriptions, codes, sub-categories, and generic categories

Transcription	Code	Sub-category	Generic category
Communicate with the rest of the team so everyone has the same information about what’s expected	Communicating with the team so everyone has the same information	Coordinating and reaffirming information	Preconditions and resources
Often you know the patient, but if you don’t then you read the patient record to get a picture	Often you know the patient, but otherwise you read the patient record and get a picture	Creating a plan for the patient and undergoing mental preparation	Planning and preparing for the expected and unexpected
It’s the planning ahead, you plan the surgical procedure. As I said, experience from this or that can happen, but then you have a plan B. Perhaps you also have a plan C as well, as it’s like … it’s people, and it can’t go wrong, you have to handle it	Managing through planning, experience, and having plans B and C.	Prioritizing and solving problems	Adapting to the unexpected

## Results

When analyzing the group interviews three generic categories emerged from the sub-categories of each professional group: preconditions and resources, planning and preparing for the expected and unexpected, and adapting to the unexpected. In each generic category, one sub-category was common and shared between the three professions: coordinating and reaffirming information, creating a plan for the patient and undergoing mental preparation, and prioritizing and solving upcoming problems as displayed in Table 3. Subsequently the generic categories with specific sub-categories representative for each profession follows.

**Table 3:** Generic categories and sub-categories pertaining to operating room nurses (ORNs), registered nurse anesthetists (RNAs) and surgeons, and those shared by the three professions

	Preconditions and resources	Planning and preparing for the expected and unexpected	Adapting to the unexpected
Shared	Coordinating and reaffirming information	Creating a plan for the patient and undergoing mental preparation	Prioritizing and solving upcoming problems
ORNs	Team coordination Having experience	Checking and having control to be prepared Taking support from roles and routines	Prioritizing and solving upcoming problems
RNAs	Maintaining focus	Creating a basic plan for work Checking and restoring	Prioritizing and solving upcoming problems
Surgeons	Having respect for the team and shared goals Having experience and competence Maintaining focus and creating space for mental rest	Creating and re-evaluating a basic plan Using guidelines and routines but with certain degrees of freedom	Prioritizing and solving upcoming problems

## Descriptions of how safe care is created shared by three professional groups

### Preconditions and resources

#### Coordinating and reaffirming information

Coordinating and reaffirming information was a sub-category that emerged as common to all three professions. If critical situations or changes in patient conditions occurred, communication was described as central to creating safe care. Having the same information was also considered essential for a well-functioning surgical teamwork. When a change of plans was called for, the ORNs often used communication with external support services such as coordinators at the OR department to convey information, get support, and obtain new equipment. When issues occurred regarding surgical instruments, the ORNs expressed communication with the surgeon to be important in order to allow prioritization and planning. The surgeons said that they interpreted communication depending on their understanding of the urgency of the situation, which helped them to prioritize. Safe communication was perceived by both ORNs and RNAs to be easier in a small workplace with shorter information paths. The ORNs said that when the team was less integrated, communication within the sub-team (e.g. ORNs and surgeons) was even more important for safe care:

“Communication is more important when the team is not well integrated. That applies to talking to each other, who does what, and what do you need help with, so you don’t get parts of the team taking it for granted that others are doing it.” (ORN)

For the RNAs, an essential precondition was the ability to get access to colleagues quickly by having a telephone nearby. From the surgeon’s perspective, communication was a prerequisite for conveying difficult moments during surgery that required an increased focus from the entire surgical team.

### Planning for the expected and unexpected

#### Creating a plan for the patient and undergoing mental preparation

An important sub-category common to all three professions emerged as creating a plan for the patient and undergoing mental preparation. In order to be mentally prepared, the professionals created a plan for the patient before the procedure. They read about the patient individually or together to create a mental model and a shared plan. From identified potential patient risks they planned what might be needed for that patient and procedure. The ORNs described how they planned and prepared for equipment adjustments prior and during a surgical procedure, based on the individual needs of both the surgeon and the patient:

“It’s based on what’s best for the patient — to ensure that the surgery will be as good as possible. Don’t hurt the patient. How does it look, what are the things you have to watch out for when you use leg support — we’re thinking about that all the time.” (ORN)

While much of the work was standardized, it was then supplemented after the ORNs had created their mental model or seen the patient. The RNAs anticipated what could happen and adjusted the plan for the patient. The plan was also communicated and structured together with the anesthesiologists, based on the anticipated scenarios. The surgeons said that in most cases they knew the patient, when this was not the case, they created a mental model of the patient and the procedure by consulting the patient record and talking to the patient:

“Often you’ll already know the patient, but if you don’t then you read the patient record and create a mental picture of them.” (Surgeon)

For the RNAs to be mentally and practically prepared clinical experience emerged as a crucial underlying prerequisite. The RNAs described a standardized routine and workflow in which information was obtained from different systems, including reported patient status by the ward nurse. Preoperatively, they also anticipated possible scenarios by inspecting and talking to the patient. Hence, possible scenarios could be identified and anticipated in advance:

“Yes, you’re prepared for it ... You might ‘read’ the patient and understand that this isn’t going to work. Like, I can see that 82-year-old Agda hasn’t had anything to drink since noon yesterday, so she’s already dehydrated…a large surgical intervention, and then when I’m positioning her I find candy under her pillow. I mean, then it’s a completely different scenario.” (RNA).

The RNAs argued, if they planned and prepared carefully in advance this was not a problem:

“Otherwise, once the process has started things just keep rolling. And you’ve, like, created this whole plan for the patient. That’s why we plan — so that won't happen.” (RNA)

### Adapting to the unexpected

#### Prioritizing and solving upcoming problems

Adapt to the unexpected, by prioritizing and solving upcoming problems was the third sub-category that emerged as common to all three professions. When unexpected issues occurred during a surgical procedure, both RNAs and ORNs said that they assessed the risks against the benefits and adapted to the situation. The ORNs expressed that prioritizing the saving of life over ensuring sterility was an important strategy for safe care:

“Sometimes you can’t scrub the patient — life is more important than ensuring sterility, and you can deal with that later. If an infection occurs, you have to treat it then. For example, we don’t scrub the urgent Cesarean sections, or the ruptured aortas when they arrive directly from the emergency room. Those aren’t the times to argue if someone comes in in white clothes, without a surgical cap and coat.” (ORN)

When problems and issues occurred during surgery, the surgeons and the RNAs expressed that problems had to be solved and it was not an option to allow things to go wrong. Surgeons described that consultation took place with more experienced colleagues or specialized hospital clinics. The problem had to be solved, and inaction was not an option.

The ORNs said that when unexpected equipment-related issues occurred, they checked the equipment, asked for a replacement or handed the problem over to a colleague and continued to focus on the surgery without being affected. The surgeons said they prioritized the interruptions that were perceived as urgent. For the RNAs, intraoperative changes in patient status were anticipated by monitoring trends in the patient’s vital signs, which allowed them to be prepared and hence respond quickly to changes. Being flexible and responsive was one of the RNAs professional skills and perceived as an inherent ability of an RNA. The RNAs explained that when facing changes or challenges they adapted to the new situation and asked for help from their colleagues. To adapt, they used previously created plans B and C, as a part of their mental model when preparing for the procedure:

“It’s the planning ahead, you plan the surgical procedure. As I said, experience from this or that can happen, but then you have a plan B. Perhaps you also have a plan C as well, as it’s like … it’s people, and it can’t go wrong, you have to handle it.” (RNA)

The surgeons perceived that working in the OR meant having to be prepared for changes and variations that sometimes contributed to a lack of focus. Unexpected urgent procedures were taken care of ad-hoc in the work process. Handling this required flexibility, adaptation, prioritization and the ability to relate to variation, interruptions and disturbances. Everyone in the care process, including staff on the wards as well as staff in the OR and recovery, had to be flexible because changes could affect everyone. Some considered variations challenging, but being able to handle a complex workday was also a positive experience which helped make the work enjoyable and stimulating:

“Or is it that they, like most of us, love their work, so it’s more a positive challenge to, like, hit the volley, I think.” (Surgeon)

## Preconditions and resources from the perspective of each profession

### ORNs’ perspectives

#### Team coordination

The ORNs described team coordination as a precondition for safe care. Familiarity with the team was described as providing security. When assisting surgeons, interaction and detection of the situation ahead were perceived as important. Cooperating with and supporting less-experienced surgeons were described as a significant part of their responsibility.

“After all, there are constantly new surgeons from different specialties who also need support, to make them feel safe and that they are moving forward, which is actually something I would say that is part of our profession. If we just stand there and wait, are grumpy, and turn our backs, the operating time extends. But when you have the flow, “a dream team” as you say, then it's wonderful.” (ORN)

The preconditions were also described as focused on the closest team members (surgeons and circulating nurse), the patient, and the assignment, as well as interacting and having a common goal.

#### Having experience

The ORNs saw experience as a resource, crucial for maintaining safe care in the OR. Being aware of one’s limitations and increased experience was said to make it easier to get a sense of the whole surgical work process. Different levels of responsibility were given to the other members of the team based on their experience. The less experience the circulating nurse had, the more responsibility was perceived to be placed on the ORN. Decision making seemed, by the ORNs, dependent on experience by making it easier to make decisions, speak up, and follow the plan. The ORNs said that if issues arose, they could always use their experience to find a solution:

“We solve problems; we see them as a challenge. Problems are there to be solved. Do the best thing possible. We now have the advantage of having so much experience that we don’t get stressed about it — we always have a plan B.” (ORN)

The ORNs also described how they gained experience by discussing and reflecting on a situation retrospectively with the other team members and learning from prior situations and decisions.

### RNAs’ perspectives

#### Maintaining focus

The RNAs said that there were many disturbances during surgical procedures. Staying focused was perceived important. To stay focused, they did not let themselves be disturbed, by conveying when it was not appropriate to interrupt and continuing with the ongoing task:

“When it comes to induction of anesthesia and the awakening, those are the sensitive phases. We can’t have people running in and out of the OR, giving a lot of information, or asking for a change. That’s when there needs to be a little more focus. Those are the situations when we’re in an extra sensitive phase, I think.” (RNA)

### Surgeons’ perspectives

#### Having respect for the team and shared goals

Respect and cooperation were considered preconditions for a well-functioning team, and the most essential prerequisite for the work in the OR:

“The team is everything. You go there to help and not to counteract each other. It has to do with respect and cooperation and all that.” (Surgeon)

Surgeons considered familiarity within the team and helping each other as a precondition for a smooth surgical workflow. Having a common goal and focusing on the patient were perceived to create the conditions for getting the job done properly. The surgeons also described a small “team within the team” comprising the operating surgeon, the assisting surgeon, and the ORN. With a well-functioning small team, they perceived themselves to be less disturbed by what was happening around them. It was important to respect the function of the team. Understanding and showing respect for one’s colleagues and recognizing that everyone was as important for the team despite having different tasks were described as prerequisites for safe care.

#### Having experience and competence

The surgeons described how they were trained from day one to handle interruptions and disturbances, which were perceived as expected and normal. They were prepared for unexpected events to occur and knew that they would have to handle the changing situation. When they were interrupted or disturbed during surgery and then continued with the primary task, it took a while to get used to these changes. However, all these abilities were linked to professional experience and would come with time:

“For that reason, I think the longer you work, the less disturbed you get, or you find some strategy for dealing with it.” (Surgeon)

As well as experience, high competence in the organization was described as an important precondition for safe care*.* Professional competence and training were important preconditions that had to be ensured by the management.

#### Maintaining focus and creating space for mental rest

Maintaining focus was considered an important ability, and the surgeons described several strategies to achieve this. When there was a high level of disturbance and noise in the OR, they tried to ignore it by staying calm, resisting, and staying in the “bell jar”. If, in spite of this, they were disturbed to the point of losing focus, they would speak up. For them to lose their focus, the interruption had to be of high urgency:

“It’s easy to say, but you have to brace yourself and stay hyper-focused. You don’t leave that state of extreme focus unless it’s something very important and relevant.” (Surgeon)

In order to maintain focus while still being able to adapt to the unexpected events that can occur during surgery, the surgeons described that they took care of unexpected issues ad hoc along the way. The strategy was to avoid cognitive overload that would consume energy. To maintain focus, they took small mental breaks; experienced surgeons said that they could do this without anyone noticing.

## Planning and preparing for the expected and the unexpected per profession

### ORNs’ perspectives

#### Checking and having control to be prepared

The ORNs considered preoperative control crucial for safety and security. To be able to plan for the expected, they described several operational checks prior to surgery including functional tests and checking of settings, the amount of gas, and availability of other material and equipment. When applicable, the marked operating area on the patient was checked, and paired organs were double-checked with radiographs and verified with the patient*.* To maintain awareness of the patient’s condition intraoperatively, the ORNs continuously observed the activity within the anesthesia team, such as looking at monitors, or calling on a colleague for support, as this was an indication of the patient’s status. Much of the preoperative preparation was performed by other ORNs or circulating nurses. For responsible ORNs to be prepared they had to check that the instruments, materials, and supplies were adequate. Counting and checking the sterile instruments and surgical tissues continuously during the procedure was another strategy described by the ORNs. To retain control, the surgeons were not allowed to pick up their own sterile instruments from the medical instrument stand. Being prepared and knowing that everything was in order before the surgeon arrived and being one-step ahead of the intraoperative process was important strategies described by ORNs. When working with new employees, the ORNs were more vigilant and prepared, as they did not know what to expect from the new colleague. However, they recognized the person’s capacity and prepared themselves mentally to provide support when needed:

“Interaction — get a sense of who the person is and give them a chance. But don’t retract those sensitive antennae — extra preparedness.” (ORN)

#### Taking support from roles and routines

When planning for the expected and unexpected, the ORNs described that they used routines and tools when preparing instruments for the procedure that existed in the OR to support their work.

“We have a lot of tools, routines, index cards, positioning guidelines — everyone has their position and knows what to do.” (ORN)

Adhering to policies and procedures, was important to reduce unnecessary interruptions or disturbances. The ORNs also described the importance of the different responsibilities of the professions in the surgical team. For example, when problems with equipment occurred, they often asked the circulating nurse for assistance as they were more skilled in handling the medical technical equipment.

### RNAs’ perspectives

#### Creating a basic plan for work

The RNAs described that they checked which OR they were placed in and the team members of the day, and then created a tentative plan of what could happen during the day*.*

“I might start by checking out the daily OR schedule in paper form, the number of procedures at this moment and what kind of procedures. Which team members, which ORN, which circulating nurse and maybe which anesthesiologist I should contact.” (RNA)

By looking at the OR schedule for the day, they could also anticipate potential changes in the schedule.

#### Checking and restoring

Another way the RNAs planned for the expected and unexpected was to conduct several pre-surgery operational checks including functional tests and checking settings and intended anesthesia equipment.

“Yes, you go through the trolley with all anesthesia equipment, locate what you need, and bring it out so it’s ready — then you can quickly see.” (RNA)

The RNAs also described how the team preoperatively checked the patient’s skin quality to prevent surgical site infections. When restoring the room after surgery, and to be prepared for acute surgery it was important to check and refill all the supplies that had been used.

### Surgeons’ perspectives

#### Creating and re-evaluating a basic plan for work

The surgeons also said they created a basic plan to be prepared and plan for the expected and unexpected.

“There’s also a basic plan, but you sort of figure out the day as it develops, and no day is like another, which is also nice — variable and revitalizing I think, compared to many boring industrial jobs.” (Surgeon)

The preparation phase started the day before, when the surgeons thought about what could be expected and how they would get things done. On the day of surgery, they checked the OR schedule again as it might have been changed. Making a rigid long-term plan was not feasible, as the plan would be verified and re-evaluated several times during the day. This was perceived as an appropriate strategy when working in an unpredictable context such as the OR.

#### Using guidelines and routines but with certain degrees of freedom

The surgeons explained that following routines and using guidelines was important for being prepared, creating a good workflow, and reducing unnecessary interruptions and disturbances during surgery. However, sometimes a deviation from routine could be necessary:

“Routines are built from standard flows. Then you also have urgent situations, but they also have routines, right? So you can know what’s coming — at a certain interval this or that will happen and we have routines for it. But in every situation, you also have to be able to improvise. It’s like those Russian ice dancers — the more they practice, the more they can improvise.” (Surgeon)

## Discussion

The main results show that to manage complexity and create safe care in the OR, the professionals shared experiences that certain preconditions and resources were crucial, including having work experience and coordinating and reaffirming information. More specifically, resilience was expressed in the professional’s capacity to prepare, respond and adapt to expected and unexpected situations. By creating a common mental model of the patient, the team established readiness to anticipate, prioritize and solve upcoming problem during the surgical procedure.

The challenges, fragility, and unpredictability of working in a CAS have been described as time- and resource constraints in the OR [13], and gaps in continuity of care, such as lack of information or communication between professionals in handover situations [24]. Why most things go right, has been proposed to be pertaining to professionals ability to accomplish their tasks by adaptations and work-arounds [6]. One common precondition for safe care was expressed by the three groups as coordinating and reaffirming information. A previous observational study, that studied how work was done, found that communication was the most common task involved in multitasking [25]. The results of the present study show that professionals described communication as an important for achieving a safe and smooth care process and may reflect the challenges that comes with working in a CAS. Speaking up may fuel resilience, from a safety culture perspective [26] members of a surgical team must have the right speak up about a perceived risk or transfer of patient information [27]. Communication has been described as comprising important transfer of information between professionals, contributing to a safe, seamless, and efficient care process in the OR. In other situations it may cause interruptions resulting in non-completion of tasks [28] or gaps in continuity of care [29] that in turn may have a negative impact on patient safety. Good outcomes have been proposed to be related to the systems adaptive capacity, the individuals, teams’ and the managements’ ability to adapt to unexpected events and changing situations, for example by using interaction and communication [30]. With a focus on how work was done in a context with variable complexity, an ethnographic study explored communication and relationship dynamics in surgical teams. Proactive and intuitive communication, silent and ordinary communication, inattentive and ambiguous communication and contradictory and high dynamic communication were identified. Different types of team collaboration were connected to the level of complexity of performed surgical procedures [31]. From the perspective of a CAS, communication is crucial for having the right preconditions to create safe care, adapt to unexpected events and creating effective team interactions and coordination. Teamwork and shared mental models are also considered crucial for patient safety in dynamic domains such as the OR [32, 33]. Communication allows a greater understanding of potential risks to develop [6] within the team, as the different professionals share their mental models [32] of the situation and ways to anticipate and be prepared to respond to system failures. A flat hierarchy seems more likely to manifest a well-functioning team communication [34].

The professionals also expressed that clinical expertise [30], experience and competence, were important individual resources to be able to plan and to meet the unexpected. According to surgeons, experience as well as organizational competence was described as an important precondition for safe care. Experienced colleagues were perceived by ORNs, as being more aware of the other team members’ capacity, competence, and need for support which made it easier to make decisions, speak up, and follow the plan. In line with other studies in the OR [19, 35, 36] the RNAs’ work experience was perceived as important for having the cognitive ability to anticipate risks, planning for the expected and unexpected, and be prepared both mentally and practically for the surgical procedure. Participants in this study had quite high mean experience which may predispose for degrees of freedom to be flexible and adapt to situations and opportunities are easier to be seen. From a theoretical perspective, experience seems a crucial component in handling the unexpected. Resilience does not merely emerge in response to specific disturbances, but develops over time from a continuous training in managing and learning from risks, stresses, and strains [37]. Mental models play a central role in individual’s behavior and sustained learning based on both one’s own experiences and those of other team members [38]. Sensemaking, retrospective and prospective learning, that is arriving at a common understanding of a situation in order to adapt to and handle it adequately evolves during communication where professionals share their expertise and knowledge [39].

When planning and preparing for the expected and unexpected, it was during these processes mental models primarily were created. This was described as collecting relevant information, anticipating potential risks, and talking to the patient. This is in line with sensemaking, a social process [30, 40], usually triggered when the team is facing an uncertain situation. It is a retrospective skill with focus on achieving plausibility, dependent on previous situational experience [41]. The same skills involved in using past experiences to find a pattern in a sensemaking process can also be used to proactively anticipate and prepare for situations that may arise. Prospective sensemaking is described as building the capacity for anticipation, which enables smooth collaboration and preparation for coping with undesired but foreseeable situations related to patient safety. Important interactions with technology in the OR have been described as prospective sensemaking, a sociotechnical process central to capturing the dynamic work in the OR supported by social and technological resources. The surgical team were shown to be constantly aware of emerging risks and were thus prepared for a rapid response [36]. Anticipating, or knowing what to expect, is also a cornerstone of resilience [16]. To some extent, planning was described differently by the three professions. The ORNs’ primary focus was on the surgical instruments, while the RNAs anticipated risks and adjusted the plan accordingly; this result is comparable to the findings of other studies of surgical teams in the OR [35, 36], and confirms the OR as a CAS [7]. In our study, the surgeons said that usually they knew the patient, but when this was not the case they planned for the patient’s care by reading the record and created a mental model. Similarly, to our results, planning [35] coordination, behavior and adaptive coordination strategies [19] have been previously described as important strategies for surgical teams to manage their tasks. The preoperative plan also showed to serve as a shared mental model for the team [19, 32] which allowed new situations to be contrasted and evaluated. In general, shared mental models have been described related to positive outcomes by creating effective teamwork [33] and minimizing preventable uncertain processes and performance [42, 43] in ad-hoc constellations of teams [42]. On an individual level, mental models can also limit professionals by using familiar ways of thinking and acting. Professionals are usually not aware of these models or potential effects on their behavior [44]. When working in a CAS it can be difficult to get a sense of the whole solely from detailed descriptions such as guidelines. Sensemaking and mental models seems to have the ability to enhance planning for the expected and unexpected. However, in a dynamic CAS such as the OR, mental models need to be shared and discussed within the team [19, 42] to avoid misunderstandings.

To be able to adapt to the unexpected, the three professional groups were unanimous in stating that prioritizing and solving upcoming problems was necessary in order to handle the unexpected. From a theoretical perspective when an unexpected event occurs, first it must be noticed, then the surgical team has to make sense of it, and then they have to do something about it [45]. To be able to adapt to unexpected events, the ORNs and RNAs described that they used previously created plans B and C, which were a part of the mental model when planning and preparing for the procedure. These results are similar to the findings of other studies in the OR context [19, 35, 36]. Having several plans appears to be a common key strategy to handle unexpected events in a CAS. However, the present study also shows the necessity of the planning phase being done carefully, as this appears to be a pre-requisite for a reflexive and quick response when unexpected events occur. From the perspective of resilience, adaptation is a central key factor that is not always about changing the plan, model, or previous approaches, but sometimes involves the readiness to modify plans to suit changing situations. Woods [46], describes this ability as being able to recognize and to stretch, extend, or change what is being done or had been planned to be done. In our study, prioritizing and solving upcoming issues was a crucial strategy as the problem had to be solved; inaction was not an option. The same strategies were also expressed in other OR studies; in order to respond to unexpected events, adaptability [33] and adaptive coordination were identified as important for safe performance, and were usually achieved through communication [19, 31, 32].

Patient safety and risk arise through variability and the managements’ ability to provide resources and pre-conditions with different degrees of freedom on which the adaptations from the surgical team are based. However, there is a need for reflection on the extent of the ability to adapt and the degrees of freedom needed in the adaptation. Resilience is often expressed as the extensibility of the system, which may result in pushing the limits for taking risks too far; this is intimately linked to exposure to risk. However, the risk of high adaptive capacity is that adaptations become normalized and signals of organizational weaknesses are masked by individual’s ability to adapt and therefore, despite system deficiencies, more difficult to be perceived by decision makers [47], balanced considerations must be considered.

### Methodological considerations

One strength of this study is the inclusion of three OR core professional groups with varied gender, age, and experience. However, the mean age and experience were both quite high, probably due to that the included OR department had quite low turnover rates among staff. This can be considered as both a strength and a limitation. On one hand, individuals with a lot of experience may contribute with more rich descriptions than those with less experience. On the other hand, perceptions from less experienced could have contributed with more variations in the phenomena of study. Transferability of qualitative results is difficult, as these results are highly dependent on the studied context. To ensure trustworthiness [48] in terms of confirmability, we have presented a selection of transcripts, codes, sub-categories, and generic categories in Table 2. To increase the credibility, interactive discussions of codes, sub-categories and generic categories took place among the authors, and quotations are presented in connection to the descriptions. Further, triangulation of sources was made of similar descriptions of the same phenomenon by the three professionals, and analyst triangulation was achieved by the research group through independent categorization. To ensure dependability, open questions were asked using an interview guide during all group interviews. The aim for choosing group interviews, instead of individual interviews was to obtain each professional group’s perceptions and experiences by dynamic group interactions. Since the OR is an unpredictable context, there was uncertainty in how many participants that could attend the planned time and day for the group interviews. The interviews were conducted at two central OR departments at one county hospital and one local county hospital in mid-Sweden by reasons as practical feasibility to obtain access to professional groups. The interviewer was an RNA, which may have affected the interpretation of the results both positively, by making it easier to interpret context-specific nuances, and negatively, by taking things for granted. As described previously, surgical teams in Sweden usually consist of six different professionals. The focus in this study was on the core professionals of the OR, including ORNs, RNAs, and surgeons. This may be considered a limitation, as not all professionals were represented.

### Conclusion

Creating safe care in the OR should be understood as a process of anticipating, planning, and preparing in order to manage challenging and complex work processes. OR staff need preconditions and resources such as having experience and coordinating and reaffirming information, to make sense of different situations. This requires a mental model, which is created through planning and preparing in different ways. Some situations are repetitive and easier to plan for but planning for the unexpected requires anticipation from experience and coordination among team members. The main results strengthen that the four abilities in the theory of resilience is used by OR staff as a strategy to manage complexity in the OR. Managing complexity seems dependent on clinical experience. Therefore, future research should focus on how to provide effective learning of effective strategies for safe practice in a complex health care environment for less experienced colleagues.

### Clinical implications

Managing complexity in the OR, being able to respond to the expected and unexpected, requires adaptive capacities such as anticipating and monitoring. Before a procedure starts surgical teams should use safety briefings to discuss potential challenges and risks and solve problems. To promote learning and to have the same goals, mental models should be shared and discussed between team members. After the surgical procedure, debriefings about what and why things went right or wrong and what could be improved may support reflective learning [34].

## Data Availability

Data are available on request for any interested researchers to allow replication of results provided all ethical and legal requirements are met according to GDPR, The General Data Protection Regulation for the European Union. Contact person, Center for Clinical Research, Dalarna, Uppsala University (dataskyddsombud@regiondalarna.se), Nissers väg 3, SE-79182 Falun, Sweden.

## Abbreviations

CAS: Complex adaptive system
OR: Operating room
ORNs: Operating room nurses
RNAs: Registered nurse anesthetists
RE: Resilience engineering
